# G-CSF prevents cerebral infarction and maintain muscle strength in experimental model of ischemic stroke

**DOI:** 10.1186/1753-6561-8-S4-P42

**Published:** 2014-10-01

**Authors:** Rafaela Aires, Brenna Lepaus Monteiro, Rayssa Florentina Scárdua, Brunelli da Rós Peruch, Lohayne Simões Barbosa, Manoel Ramos Penha, Marco Cunegundes Guimarães, Breno Valentim Nogueira

**Affiliations:** 1 EMESCAM/UFES, Vitoria, Brasil; 2 Federal University of Espirito Santo, UFES, Vitoria, Brasil; 3 Postgraduate Program in Biotechnology Federal University of Espirito Santo - PPGBIOTEC/UFES, Vitoria, Brasil

## Background

Cerebral infarction is an ischemic stroke resulting from a disturbance in the blood vessels supplying blood to the brain, being the leading cause of physical and cognitive disabilities in adults [1]. The currently approved administration of thrombolytic agents is effective only within about the first 3 hours poststroke [2]. Recent studies have demonstrated that administration of growth factors can reduce stroke size or functional deficits [3]. Among the factors, the granulocyte colony-stimulating factor (G-CSF) demonstrated ability to promote differentiation of hematopoietic cells, as well as neurogenesis and promoting formation of new synapses [4,5]. Therefore, the aim of this study was to evaluate if the protective role of G-CSF in cerebral ischemia is associated with the maintenance of muscle strength.

## Methods

Swiss webster mice (*Mus musculus*) males (n = 16), weighing approximately 30 g, underwent global cerebral ischemia. Was occluded common carotid arteries for 80 minutes, and after this period the blood flow of the common carotid artery was released, while the arterial blood supply to the left remained interrupted. The stroke animals received vehicle (5% glucose solution) or were treated with G-CSF at a dose of 100 mg /kg /day, administering it after 24 hours of treatment. All the *experimental *procedures were *performed in accordance withNational Institutes of Health (NIH) guidelines, and study protocols were previously approved by the Institutional Animal Care and Use Committee (CEUA Protocol # 011/2011)*. The measurement of the strength of the mice was performed in the pre and post surgery through software coupled to a force transducer. The quantification of the area of cerebral infarction using 2,3,5-triphenil tetrazolium chloride was established by morphometric analysis using Image J program (NIH). The data are presented as means ± SEM. Statistical analysis was performed using Student's *t *test for comparison of groups using the software Prism^® ^5.0 (GraphPad, San Diego, CA, USA). p values <0.05 were considered to be statistically significant.

## Results and conclusions

A significant increase in the number of circulating leukocytes in the animals treated with G-CSF (= AVE + vehicle 2,550 ± 283/mm3 vs. G-CSF + AVE = 15,650 ± 1,294/mm3, p <0.01) was observed. The strength after surgery was significantly higher (p <0.05), in the group treated with G-CSF (88 ± 4 g; *t *value = 0.0473) when compared with vehicle group (71 ± 5 g). The areal extent of cerebral infarction was significantly lower (p <0.05) in animals treated with G-CSF (0.205 ± 0.03 cm^2^; Student *t *value = 0.0331) compared to the control group (0.401 ± 0.07 cm^2^).

Our results demonstrate the neuroprotective effect of G-CSF in mice undergoing ischemic brain, thereby contributing to the reduction of neurofunctional impairment caused by stroke, as the maintenance of strength in the treated group.
